# *Cryptosporidium *and *Strongyloides stercoralis *infections among people with and without HIV infection and efficiency of diagnostic methods for *Strongyloides *in Yirgalem Hospital, southern Ethiopia

**DOI:** 10.1186/1756-0500-3-90

**Published:** 2010-04-01

**Authors:** Amde Getaneh, Girmay Medhin, Techalew Shimelis

**Affiliations:** 1 Hawassa Regional Laboratory, Hawassa, Ethiopia; 2 Aklilu Lemma Institute of Pathobiology, Addis Ababa University, Addis Ababa, Ethiopia; 3 Department of Medical Laboratory Science, Hawassa University, Hawassa, Ethiopia

## Abstract

**Background:**

Cryptosporidiosis and strongyloidiasis have been reported to be associated with HIV/AIDS. The present study was designed to determine the prevalence of *Cryptosporidium *and *Strongyloides stercoralis *infections among people with and without HIV infection and also assess the efficient methods for detection of *Strongyloides*.

**Findings:**

A cross-sectional study was conducted in Yirgalem Hospital, southern Ethiopia from March, 2007 to October, 2007. Demographic data and stool samples were collected from 384 individuals (192 from each HIV serogroup). Samples were processed using the modified Ziehl-Neelsen technique for detection of *Cryptosporidium *species. Stool samples were also processed using the direct saline mount, the formol-ether and the water-emergence techniques for diagnosis of *S. stercoralis*. The prevalence of *Cryptosporidium *and *S. stercoralis *among HIV infected individuals was 25% and 12.0%, respectively. HIV positive individuals had significantly higher rate of infection with *Cryptosporidium *(OR = 15.7; 95% CI 5.5 to 44.5) and *S. stercoralis *(OR = 6.4; 95% CI 2.2 to 18.9). Among the three diagnostic methods, the larvae of *S. stercoralis *were more efficiently detected by the water-emergence technique.

**Conclusions:**

In this study, the prevalence of *Cryptosporidium *and *S. stercoralis *infections was significantly higher among people with HIV/AIDS. Educating HIV infected individuals to prevent acquisition of *Cryptosporidium *infection and screening for *S. stercoralis *using the water-emergence technique is likely to be helpful.

## Background

As the number of people living with human immunodeficiency virus (HIV) continues to increase, Acquired Immunodeficiency Syndrome (AIDS) remains to be a major global health priority and among the leading causes of death [1]. Diarrhea is one of the most common AIDS-related illnesses causing a significant morbidity and mortality in HIV-infected patients [2]. Viral, bacterial and parasitic infections frequently cause diarrhea though that of parasitic origin is prominent in patients with AIDS in developing countries [3].

*Cryptosporidium *is one of ubiquitous protozoan parasites with worldwide distribution and it causes diarrhea in humans and animals. Transmission of this parasite is mainly through fecal-oral route, as well as through drinking contaminated water, person-to-person spread and contact with infected animals [4]. In immunocompetent individuals, *Cryptosporidium *usually causes a self-limiting diarrhea, whereas in immunodeficient patients it may cause a severe, chronic and progressive gastroenteritis. Because no effective therapy is available for cryptosporidiosis, prolonged diarrhea may lead to dehydration, wasting and frequently death [5,6]. A number of studies have determined prevalence of cryptosporidiosis among HIV-positive patients and showed results differing quite markedly from one another and ranging from 0 to 100% [6].

The intestinal nematode *Strongyloides stercoralis *is also another important human parasite with gastrointestinal manifestations. Tens of millions of persons are infected worldwide although no precise estimate is available [7]. Transmission of *S. stercoralis *is mainly through skin contact with soil contaminated with infective larvae [8]. In immunocompetent individuals, infection with *S. stercoralis *is usually asymptomatic or causes mild to moderate abdominal symptoms [7]. In immunosuppressed patients, however, hyperinfection and dissemination of worms to ectopic sites (e.g. brain) causes severe illness [9] leading to high mortality (up to 87%) [8]. The recommended treatment for *S. stercoralis *is either ivermectin (200 μg/kg body weight in a single dose) or albendazole (400 mg daily for 3 days) [10].

With the increasing number of individuals with HIV/AIDS, investigating the influence of HIV-induced immunodeficiency on the epidemiology and outcome of *S. stercoralis *infection is essential. Indeed, the prevalence of *S. stercoralis *is usually underestimated because the parasite presents diagnostic challenges. Infections usually do not manifest noticeable characteristic symptoms nor do conventional diagnostic techniques efficiently detect the parasite [8]. The direct saline mount, for instance, is known to suffer from lack of sensitivity despite its wide and routine use for the diagnosis of *Strongyloides *in our settings. Therefore, the need for more efficient methods that improve diagnosis particularly in those at risk to develop the severe form of the disease is warranted. This study was designed to determine the prevalence of *Cryptosporidium *and *S. stercoralis *infections among people with and without HIV infection and also assess the efficient methods for detection of *Strongyloides*.

## Methods

A hospital based cross-sectional study was conducted to determine the prevalence of *Cryptosporidium *and *S. stercoralis *infections in Yirgalem Hospital, southern Ethiopia from March, 2007 to October, 2007. The outpatient departments of the hospital were serving about 175 patients per day; 25 of whom were admitted in different wards and the mean inpatient stay was 7.5 days [11]. This hospital routinely delivers HIV counseling and testing to serve people seeking to know their HIV status and to enable health care providers offer specific medical services. People who test HIV-positive were investigated on regular basis to monitor their disease status. In 2007, the adult point prevalence of HIV infection in South Nations and Nationalities People Region (where the hospital is located) was estimated to be 1.4% [12].

The study participants consisted of consecutive clients who test HIV-positive and HIV-negative in the hospital during the study period and volunteered to have stool investigation. Individuals treated for any intestinal parasites during the month prior to the study and those under the age of 15 years were excluded. In total, 384 clients (192 from each serogroup) participated in this study. HIV infected patients were classified as 1 through 4 clinical stages according to the criteria of the World Health Organization Clinical Staging of HIV/AIDS [13].

Study subjects were interviewed about demographic factors using structured questionnaires. A single fresh stool sample was collected from each participant and processed for microscopic examination of *Cryptosporidium *and *S. stercoralis *according to the described methods [14].

More specifically, stool samples were processed using the modified Ziehl-Neelsen technique to detect *Cryptosporidium *species. In this technique, air dried stool smears were fixed in methanol for 3 minutes, stained by carbol fuchsine for 15 minutes, decolorized with 1% acid alcohol for 15 seconds, and counter stained with 0.5% methylene blue for 30 seconds. Stained smears were air dried and examined microscopically for oocyst of *Cryptosporidium *using 100× objective.

For detection of *S. stercoralis*, samples were processed using three different parasitological techniques: namely, the direct saline mount, the formol-ether concentration and the water-emergence technique. In the direct saline mount, fresh stool samples were emulsified with 0.85% physiological saline and examined microscopically for larvae of *S. stercoralis *using 10× objective. In the formol-ether technique, about 1 gram of faeces was emulsified in 10% folmol-water and then strained to remove large faecal particles. Sieved suspension was mixed with diethyl ether, centrifuged at 3000 rpm for 1 minute. The sediment was examined microscopically for the larvae using 10× objective. In the water-emergence technique, a central depression was made in fresh stool specimen and filled with warm water (about 37°C). The specimen was incubated at 37°C for an hour during which time larvae crawl out of the faeces and migrate into the warm water. Some of the water was transferred to a slide and examined microscopically. A given participant in the study was classified as positive for *S. stercoralis *whenever the larvae were recovered by at least one of the methods.

Data entry and analysis was performed using SPSS Version-15 and descriptive summary was presented in terms of mean, range and proportions depending on the scale of the variable. Binary logistic regression analysis was used to assess the crude effects of demographic characteristics and HIV status on a given outcome variable. A given statistical test was reported significant whenever it resulted in a p-value < 0.05. The strength of association between a given exposure and binary outcomes was measured using odds ratio and its corresponding 95% confidence interval. Cohen's kappa [15] was calculated to show the degree of agreement between diagnostic methods of *S. stercoralis *using STATA Version-10.

The study was approved by the Ethics Committee of the South Nations and Nationalities People Region Health Bureau and the Yirgalem Hospital. Participation was fully voluntary and all study subjects gave informed written consent. Physicians managed those individuals found to be infected with any intestinal parasites.

## Results

A total of 384 individuals participated in the present study. Most of the participants were urban dwellers (69.0%), male (58.3%), and in the age range 30-39 years (32.8%). Participants with HIV infection were 50%. The mean age of HIV infected participants was 34.8 years (range 15- 67 years; SD 9.4) compared to 30.1 years (range 15- 65 years; SD 12.9) in HIV un-infected clients. The male to female ratio was 1.3:1 in HIV positive and 1.5:1 in HIV negative clients. Majorities of HIV infected participants were in the clinical stage four (42.2%) and in the clinical stage three (40.1%).

The rates and unadjusted odds ratios of *Cryptosporidium *and *S. stercoralis *infections stratified by demographic factors and HIV status are summarized in table 1. The prevalence of *Cryptosporidium *infection was 25% (48/192) and 2.1% (4/192) among participants with and without HIV infection, respectively. Moreover, *S. stercoralis *were detected in 12.0% (23/192) and 2.1% (4/192) of participants with and without HIV infection, respectively. HIV infected participants had significantly higher risk of *Cryptosporidium *[odds ratio (OR) = 15.7; 95% CI 5.5 to 44.5] and *S. stercoralis *infection (OR = 6.4; 95% CI 2.2 to 18.9) compared to HIV un-infected participants. Two cases of co-infection with both parasites were observed among HIV infected participants. No case of *S. stercoralis *infection was detected among HIV infected participants in the clinical stage 1. HIV positive participants categorized in different clinical stages had no statistically significant difference in *Cryptosporidium *infection rate.

**Table 1:** Unadjusted effects of HIV status and demographic factors on the positivity of *Cryptosporidium *and *Strongyloides stercoralis*, Yirgalem Hospital, southern Ethiopia, 2007.

Characteristics	Total (%)	Type of parasite					
		
		*Cryptosporidium *species			*Strongyloides stercoralis*		
		
		Positive (%)	Crude OR (95% CI)	P- value	Positive (%)	Crude OR (95% CI)	P- value
Residence							
Rural	119 (31.0)	5 (4.2)	1		3 (2.5)	1	
Urban	265 (69.0)	47 (17.7)	4.9 (1.9-12.7)	0.001	24 (9.1)	3.9 (1.1-13.0)	0.03
Age							
< 20	46 (12.0)	2 (4.3)	1		1 (2.2)	1	
20-29	107 (27.9)	14 (13.1)	3.3 (0.7- 15.2)	0.12	5 (4.7)	2.2(0.3-19.4)	0.47
30-39	126 (32.8)	21 (16.7)	4.4 (1.0- 19.6)	0.05	12 (9.5)	4.7(0.6-37.5)	0.14
40-49	69 (18.0)	10 (14.5)	3.7 (0.7- 17.9)	0.10	7 (10.1)	5.1(0.6-42.8)	0.14
≥ 50	36 (9.4)	5 (13.9)	3.6 (0.7- 19.5)	0.15	2 (5.6)	2.6(0.2-30.4)	0.44
Sex							
Male	224 (58.3)	25 (11.2)	1		15 (6.7)	1	
Female	160 (41.7)	27 (16.9)	1.6 (0.9-2.9)	0.11	12 (7.5)	1.1 (0.5-2.5.)	0.76
HIV status							
HIV negative	192 (50)	4 (2.1)	1		4 (2.1)	1	
HIV positive	192 (50)	48 (25)	15.7 (5.5-44.5)	0.000	23 (12.0)	6.4 (2.2-18.9)	0.001
Stage 1	4 (2.1)	1 (25)	15.7 (1.3-185.3)	0.03	0	-	
Stage 2	30 (15.6)	8 (26.7)	17.1 (4.8-61.4)	0.000	5 (16.6)	9.4 (2.4-37.3)	0.001
Stage 3	77 (40.1)	21 (27.3)	17.6 (5.8-53.5)	0.000	7 (9.1)	4.7 (1.3-16.5)	0.02
Stage 4	81 (42.2)	18 (22.2)	13.4 (4.4-41.1)	0.000	11 (13.6)	7.3 (2.3-23.9)	0.001

Among all participants, 7.0% (27/384) were positive for *S. stercoralis *by at least one of the three diagnostic methods. Of the total positive samples, 85.2% (23/27) were recovered by the water-emergence technique, 55.6% (15/27) by the direct saline mount, and 51.9% (14/27) by the formol-ether technique. This shows that the contribution of positive diagnosis to the domain of positives by at least one of the three techniques was significantly higher by the water-emergence technique compared with the direct saline mount (p = 0.033) and the formol-ether concentration technique (p = 0.020). However, there was no statistically significant difference between the direct saline and the formol-ether techniques (p = 0.564) (Table 2).

**Table 2:** Comparative analysis of three methods for the diagnosis of *Strongyloides stercoralis *in 27 positive stool samples from Yirgalem Hospital, southern Ethiopia, 2007.

Parasitological diagnostic technique	Positive for *S. stercoralis*		McNemar's Chi-square (df = 1)	One sided P- value
			
	Number	Percent		
Water- emergence	23	85.2	4.57	0.033
Direct saline mount	15	55.6		
				
Water- emergence	23	85.2	5.40	0.020
Formol-ether	14	51.9		
				
Direct saline mount	15	55.6	0.33	0.564
Formol-ether	14	51.9		

Evaluation of agreement among the diagnostic methods for *S. stercoralis *using kappa statistic showed that the direct saline mount and the formal-ether concentration had strong diagnostic agreement (Kappa = 0.89; 95% CI 0.71 to 0.96) and they both resulted in a very low rate of recovery. The water-emergence technique had lower level of agreement with the direct saline mount (kappa = 0.61; 95% CI: 0.42, 0.76) and with the formal-ether concentration technique (kappa = 0.58; 95% CI: 0.38, 0.73) but it yielded higher rate of recovery.

## Discussion

We determined the prevalence of *Cryptosporidium *and *S. stercoralis *infections among participants with and without HIV infection. In this study, the prevalence of *Cryptosporidium *and *S. stercoralis *infection among HIV infected individuals was 25% and 12%, respectively. A comparable rate of *Cryptosporidium *infection was reported among HIV-infected individuals in Hawassa (southern Ethiopia) (20.1%) [16]. However, a lower rate of *Cryptosporidium *infection (3.1%) was previously reported from Jimma (southwest Ethiopia) [17]. Similar rates of *S. stercoralis *infection were also reported in studies from Hawassa (12.6%) [16], Jimma (7.8%) [17], and Gondar (northwest Ethiopia) (10.7%) [18].

In this study, HIV status was significantly associated with *Cryptosporidium *and *S. stercoralis *infections. Increases in odds of *Cryptosporidium *and *S. stercoralis *infections were about fifteen-fold and six-fold, respectively, among HIV positive individuals compared with HIV negatives. In comparison, rates of *Cryptosporidium *infection were about three-fold among HIV infected individuals in Italy [19] and ten-fold in south India [20]. Further, HIV infection raised the odds of cryptosporidiosis about forty-four times in Ugandan children [21]. *Cryptosporidium *infections also occurred exclusively among HIV positive individuals, with rates ranging from 3.1% to 85.9% [17,22-24]. Similarly, studies have showed significant predominance of *S. stercoralis *among HIV infected individuals, with increases ranging from about four-fold to twenty-three-fold [16,17,25,26]. Thus, in spite of varying strength of associations, findings seem consistent that HIV infected persons more likely have higher risk of *Cryptosporidium *and *S. stercoralis *infections.

In the present study, the association between *Cryptosporidium *and course of HIV infection was not evident. This is possibly because clinical staging may not be accurate to show the level of patient immune status. Studies have reported that the host immune status is one of the important determinants to influence *Cryptosporidium *[16,19,23,27] and *S. stercoralis *infection [16]. Patients with CD4 T-cell count less than 200 cells/μl of blood are in excess risk of having these infections [16]. This may be due to the adequacy of lower doses of *Cryptosporidium *to initiate infection in immunocompromised hosts [28] as well as the persistence of established infection for longer time within these hosts, which modifies incidence and course of infection [6]. The unique ability of *S. stercoralis *to replicate within the host (autoinfection), similarly, results in persistence. And the failure of immune system to control larvae emerging from the auto-infective cycle may lead to uncontrolled multiplication (hyperinfection) in immunosuppressed patients [10]. However, impact of HIV-induced immunodeficiency to influence either prevalence or outcome of *S. stercoralis *infection is not well defined. In the present study, although the possibility for differential occurrence of important risk factors was not ruled out, it may be an alteration in immune status resulting in increased rates of *Cryptosporidium *and *S. stercoralis *infections among people with HIV.

Because intermittent excretion of fewer *Strongyloides *larvae are usual, examinations of single stool samples using conventional techniques fail to detect larvae in up to 70% of cases. Thus, repeated examinations of stool specimens have been recommended to improve the chances of recovering the parasite. Diagnostic sensitivity increases to 50% with 3 stool examinations and can approach 100% if 7 serial stool samples are examined [8]. However, performing repeated stool examinations seem impractical in our settings where testing adherence of clients and sustainability of diagnostic services have difficulty to be ensured. In this study, the water-emergence technique was found to be about 1.5 times more efficient to detect *S. stercoralis *than either the direct saline mount or the formol-ether concentration technique. Therefore, this method may preferably be used for screening high risk individuals and those suspected of infection but found to be negative by less sensitive methods.

However, the results of this study should be interpreted with cautions because of its methodological limitations. Firstly, as any hospital based cross-sectional study, it undoubtedly introduces selection bias making generalization impossible. Secondly, the study failed to rule out the possibility that the two groups were unmatched in light of the most important risk factors. Thirdly, attempt was not made to examine multiple stool samples; thus, failure to detect larvae in the stool samples did not necessarily indicate the unequivocal absence of the infection. Last, the methods employed for the diagnosis of *S. stercoralis *did not include methods of choice (e.g. Baermann technique) against those comparisons of the water-emergence technique would have been imperative.

In conclusion, the rates of infections with *Cryptosporidium *and *S. stercoralis *were significantly higher among HIV infected individuals compared with HIV negative group. Educating HIV infected individuals to prevent acquisition of *Cryptosporidium *infection and screening for *S. stercoralis *using the water-emergence technique is likely to be helpful.

## Competing interests

The authors declare that they have no competing interests.

## Authors' contributions

AG designed and carried out the laboratory work; TS and GM performed the statistical analyses; AG and TS interpreted the result; all authors contributed to the write up; read and approved the final manuscript.
